# Auditory and Language Development Assessment of Newborns Aged One to Four Years Exposed to Gestational Zika Virus Infection: A Case Series

**DOI:** 10.3390/ijerph18126562

**Published:** 2021-06-18

**Authors:** Liora Gonik, Amanda Tupinambá da Fonseca Oliveira, Paula Silva de Carvalho Chagas, Jaqueline da Silva Frônio

**Affiliations:** Graduate Program of Rehabilitation Sciences and Physical and Functional Performance, Faculty of Physiotherapy, Universidade Federal de Juiz de Fora, Juiz de Fora 36038-330, MG, Brazil; gonikdias@hotmail.com (L.G.); tupinambah.fisioterapia@gmail.com (A.T.d.F.O.); pscchagas@gmail.com (P.S.d.C.C.)

**Keywords:** Zika virus, children’s language, hearing, hearing loss, child development

## Abstract

The known neurotropism of the Zika virus (ZikV) suggests that auditory organs and their neural pathways may be affected by prenatal Zika infections. Among the possible manifestations are audiological and language disorders, but so far, the data in the literature are inconclusive. Objective: To describe early and late hearing disorders in children with Congenital Zika Virus Infection (CZVI) and evaluate the language development of this population between 14 and 47 months of age and its possible correlation with the alterations found in auditory exams. Methods: Longitudinal, prospective, observational study of newborns born in Juiz de Fora and its macroregion with confirmed diagnosis of ZikV infection during pregnancy. Participants were examined from one to four years of age for hearing using the transient otoacoustic emissions (TOAE) test, immittance testing and brainstem auditory evoked potential (BAEP), and language using the Bayley Scales of Infant Development—Third Edition (Bayley III). Results: 15 participants were included; eight (53.33%) presented alterations in at least one of the hearing tests, one had an early loss (6%) of sensorineural origin, and seven (46.67%) had a poor language performance. In the three (20%) participants whose audiological exams were altered, there was language impairment, and two (13.33%) participants had extensive malformations in the central nervous system (CNS), presented language delay, and hearing exams were within normality. Conclusion: Infants and preschoolers with CZVI may present early neurosensory loss and late hearing loss with fluctuating character. Even if there were no significant association between the audiological exams results and the Bayley III performance, in the present sample, language development was below expectations for the age in the participants who had alterations in the three audiological exams, when there is early hearing loss or extensive lesions to the CNS. The results reinforce the importance of audiological examinations, especially the BAEP morphological and auditory threshold, in monitoring cases of CZVI until at least three years of age.

## 1. Introduction

A Public Health Emergency of International Concern was declared by the World Health Organization in February 2016 [1] due to the notification and response of the Brazilian government to the increase in the prevalence of microcephaly and other changes in the central nervous system related to Congenital Zika Virus Infection (CZVI) [2,3]. This event is one of the most complex and impacting epidemics in the history of public health in Brazil [4] and has allowed the design of a new health condition of what is now known as the Congenital Zika Syndrome (CZS). Although microcephaly is recognized as the main and most worrying clinical outcome of this condition [5], it is important to highlight the need to investigate other possible impairments in infants and children whose mothers had Zika virus (ZikV) infection during pregnancy, even in asymptomatic cases at birth [6].

The known neurotropism of the ZikV [7] suggests that the auditory organs and their neural pathways can be affected, leading to both morphological and functional impairments, especially in prenatal infections. There are other infections that, if they occur during the gestational period, have a greater potential to cause changes in fetal development. They are currently known by the acronym SCORTCH (syphilis, cytomegalovirus, others, rubella, toxoplasmosis, chicken pox, herpes simplex, and blood-borne viruses) [8]. Among the changes resulting from such congenital infections is hearing loss, which is an outcome in part of samples already described in the literature. Its incidence varies according to the causative agent. Congenital toxoplasmosis affects 14 to 26% of the infected newborns and leads to sensorineural hearing loss of a deep, unilateral, or bilateral degree [9,10]. Congenital rubella may lead to moderate to severe bilateral sensorineural loss in 12 to 19% of cases and severe to profound bilateral loss in 7.4% of cases [11]. In congenital syphilis, sensorineural hearing loss ranges from 25 to 38%, and there may be periods of exacerbation with fluctuations of hearing, accompanied or not by vestibular changes [12]. Congenital cytomegalovirus leads to hearing loss in approximately 25% of cases with neuroimaging changes and in 8% of patients with a normal neuroimaging result [9,12]. These data show that hearing loss is frequent in cases of gestational infections, and that some of these losses may not be identified in the first months of life, especially in cytomegalovirus infections, or may occur later, as with syphilis [11].

In a retrospective study, a complete assessment of hearing function in a series of 70 children with microcephaly and laboratory evidence of CZVI showed that four (6%) infants had sensorineural hearing loss. The examination of otoacoustic missions (OAS) is performed as a neonatal screening test and the frequency-specific brainstem auditory evoked potential (BAEP) is performed one month later to confirm the loss [13]. However, the reports may underestimate the incidence of hearing loss, because only children with microcephaly were included in those studies and the participants underwent hearing tests only in the first year of life.

The review on hearing findings associated with ZikV infection by Barbosa et al. [14] concludes that most of the children evaluated were submitted only to hearing screening tests with the otoacoustic emissions test and/or BAEP, and that audiological studies of children infected in the prenatal period by the ZikV are very heterogeneous regarding the performance of the tests and their methods, leading to a difficulty in proposing an optimized protocol for a topographic and morphological diagnosis of the most frequent type of hearing loss in the presence of this health condition. Regarding changes in language development, Rosa et al. [15] carried out a systematic review study. It is the most recent review on the subject, and the author found four papers that addressed this aspect: Flor et al. [16], Avelino et al. [17], Alves et al. [18], and Carvalho et al. [19]. The first three studies only performed developmental screening tests (Denver II) and the fourth study [19] used a more complete language scale (Bayley III scale), but only in infants diagnosed with Cerebral Palsy likely due to CZVI with one year or more of age. None of these studies discussed the possible association between the presence of audiological disorders and language development.

Considering the lack of prospective longitudinal studies that thoroughly investigate hearing and language in a population of preschoolers proven to be exposed to Gestational Zika Virus Infection, the objective of the present study is to describe early and late hearing disorders in children with CZVI and evaluate the language development in this population between 14 and 47 months of age and its possible correlation with the alterations found in auditory exams.

## 2. Materials and Methods

A longitudinal, prospective, observational study was carried out. This is part of a larger project entitled: “Gestational infection by Zika Virus: child development from zero to seven years of age, environmental context and epidemiological profile”, which was approved by the Ethics Committee in Research on Human Beings of the Federal University of Juiz de Fora (UFJF), Juiz de Fora, Minas Gerais, Brazil, under opinion np. 2,001,169.

### 2.1. Sample

Newborns born from November 2015 to March 2018 in Juiz de Fora and its macroregion, who had a confirmed diagnosis of ZikV infection during pregnancy, were included. Cases that presented genetic syndromes, progressive diseases or congenital toxoplasmosis, rubella, cytomegalovirus, HIV, herpes, and syphilis were not included.

Of the 19 potential participants in Juiz de Fora and its microregion [20], 15 preschoolers, aged one to four years, representing 79% of all cases with GZVI, were included in this study. As for the other four cases of GZVI in this period, one parent refused to participate in the study and it was not possible to contact the other three using the data provided by the Department of Epidemiological Surveillance, even after several attempts.

The diagnosis of GZVI was made during pregnancy on the participants’ mothers using RT-PCR for cases where blood collection was performed until the fourth day of the clinic period (rash, itching, and low fever) [21,22], or the antibody test (IgG and IgM) for Zika, when it was not possible to perform the RT-PCR. The data were made available to the research team by the Department of Epidemiological and Environmental Surveillance of Juiz de Fora and its surrounding region.

### 2.2. Evaluation

Participants were assessed longitudinally for hearing and language at different times between 14 and 47 months using the instruments and tests described below. 

To assess hearing, three instruments were used: the transient otoacoustic emissions test (TOAE), immittance testing, and the brainstem auditory evoked potential (BAEP). The TOAE recording was performed by the Otodynamics Otoport Lite equipment, which uses the non-linear click stimulus predominantly in the frequency range between 1000 to 4000 Hz, with an intensity of 64 dB pe NPS. The emission analysis criterion was a 6 dB SNR signal/noise ratio in three out of five tested frequency bands. A total of 260 sweeps of 16 stimuli were used. The maximum test time was 300 seconds, and the recording frequency bands of the device were 1.0 1.5, 2.0, 3.0, and 4.0 kHz. The parameters used to consider the presence of responses were reproducibility above 50%, as well as signal-to-noise ratio >3 dB at 1.0 and 1.5 kHz, and >6 dB at 2.0, 3.0, and 4.0 kHz. The protocol was used to allow the analysis of pass/fail criteria for each of the five frequency bands tested in three present frequency bands [23]. The presence of TOAEs indicates that the preneural cochlear receptor mechanism is capable of responding to sound in a normal way, which has the greatest clinical value.

Immittance testing was performed using an Interacoustics Immittance Meter, model AT235h. The tympanometric curves were classified into: curve A (normal), with a single peak and pressure between −100 and 100 daPa and volume from 0.28 to 2.5 mL; air curve (reduced amplitude) with a reduced compliance peak, below 0.28 mL; curve Ad (increased amplitude) with a double peak, increased compliance above 2.5 mL, and curve B, or flat, without pressure peaks. In a type C curve, the peak shifted to negative pressure below −100 daPa. The acoustic reflex was considered present when present in all frequencies [24,25]. The immittance testing consists of verifying the compliance or mobility of the tympanic–ossicular system due to the pressure variation introduced in the external auditory canal. This test provides quantitative information regarding the presence of fluids in the middle ear, mobility of the tympanic–ossicular system, and volume of the external auditory canal, which is important for topodiagnosis [26]. Secretory otitis media (SOM) is characterized by curves B and C [24]. 

For BAEP registration, the Contronic’s Evokadus equipment was used. In individuals with normal hearing: stimulation at high intensities between 75 and 90 dBHL, latency of wave V of 5.5 to 6.0 ms, increasing approximately 2.0 ms until the threshold. The latency of wave I is 1.5 ms, of wave III is around 3.5 ms, and the interpeak intervals are approximately 2.0 ms. At lower intensities, the latencies of all waves increase and wave I can be undetectable. The threshold is defined by the lowest intensity at which the V wave can be replicated [27]. It is an electrophysiological examination by which it is possible to obtain information about the functioning of the auditory system up to the brainstem, which is ideal for assessing babies and children who are unable to respond for behavioral assessment. 

Language development was assessed using the Bayley Scales of Infant Development—Third Edition; the version was translated and adapted to Brazilian Portuguese (Bayley III) [28]. The Bayley III is a standardized and appropriate instrument for assessing the infant development of children aged one to 42 months (with and without pathologies), enabling a possible early intervention in the case of delayed child development. The language scale has 97 items, which are subdivided into receptive language (49 items) and expressive language (48 items). The scoring of scales is based on the performance or not of the proposed activities, crediting one point or zero, respectively. The calculation of the final score of the scale is performed through the sum of all items scored in the evaluation with the results of testing at previous ages, resulting in a raw score of each subscale. The gross score value of each subscale is converted into standardized points to obtain the scaled score (SS) and the composite score (CS) for the language scale, which reflects the sum of performance in the two subscales that compose it. To analyze the language development data of the participants in the present study, SS and CS were used as continuous variables and their categorizations were as described below. For SS categorization, the one recommended by the technical application manual of the Bayley III Language Scale was used, where values lower than seven are considered below the expected value for the age. The cutoff point recommended by [29] was used, where adequate performance is CS ≥ 85 and reduced performance: CS < 85.

### 2.3. Procedures

Participants were recruited from the list of live newborns notified by the SINASC (Live Birth Information System)/RESP (Public Health Event Records)/SIRAM (Child Care with Microcephaly Record System), provided by the Epidemiological and Environmental Surveillance Department of Juiz de Fora and its surrounding region. All confirmed cases of GZVI were invited to participate through their parents or legal guardians. The first contact was by phone call by one of the team members responsible for the data collection of the study. In the case of fulfilling the eligibility and acceptance criteria regarding participation, a date was scheduled to attend the data collection site (Laboratory for the Evaluation of Child Development-LADIN, at the Faculty of Physiotherapy at UFJF, Juiz de Fora, Minas Gerais, Brazil). On the day scheduled for the first evaluation, the parents signed an informed consent (IC), and the necessary information was collected for the characterization of the participants and the examinations and treatments carried out until that moment. That same day, Bayley III was also applied in a complete way by a trained team. When realizing that the child was tired, the team interrupted the assessment and scheduled a new date to resume the test application, within a maximum period of seven days. The audiologic exams (OEAT, immittance testing, and BAEP) and the complementary questionnaire (aiming to survey other risk factors for hearing loss and previous exams) were performed within a maximum period of seven days after the application of the Bayley III in a private clinic specialized in otorhinolaryngology (Clínica GonikDias, Juiz de Fora, Minas Gerais, Brazil) at no cost to the participants. Parents or guardians were able to monitor all assessments, as long as they did not interfere with the participant’s performance. 

The evaluations of the participants were programmed with a six-months interval until the age of 48 months, which included a series of complete assessments (Bayley III and audiological tests). Those who attended at least one of these complete assessments were included in the present sample. The majority of participants were older than two years of age upon conduction of the first audiological tests. In each attendance, a follow-up questionnaire was filled out containing information about the treatments and possible complications that occurred during the period, and an appointment was made for the subsequent evaluation. When the participant did not show up at the scheduled date and place, a new telephone contact was made, and the assessment was rescheduled to the closest possible date.

All children were submitted to hearing screening with the three scheduled tests in order to observe the results in the tests and an equivalence regarding the involvement or not of the auditory pathways. The child was considered a “pass” in the screening when it showed adequate responses in the three tests. The failure in any of the audiological tests, even with a response in the normal morphological BAEP and a threshold at the minimum level of 30 dBHL, implied referral for differential diagnosis and possible treatments with an otolaryngologist. On the Bayley III, when the CS was below 85 (reduced performance), the participants were referred to speech therapy services. OEAT, immittance testing, BAEP, and Bayley III were repeated every six months.

### 2.4. Statistical Analysis

The data were stored and analyzed using the Statistical Package for the Social Sciences (SPSS), version 22.0. To present the sample results, the data were organized into three age groups that had a greater number of attendances in the scheduled evaluations and exams: 30 to 31 months, 36 to 38 months, and 40 to 42 months. The interval between one assessment and another for each participant was at least six months. One of the participants had not yet reached the age of 30 months by the end of the study, and the data from its exams are described separately here and were excluded from statistical analyses of performance in tests and possible associations. 

Descriptive statistics were performed with values of absolute and relative frequency for categorical variables and mean and standard deviation for continuous variables. For the comparison of two categorical variables, the Chi-square test or the Fisher’s exact test was used. To compare a numeric and a categorical variable, the Mann–Whitney test was used due to the sample size. For all analyses, the significance level of α = 0.05 was considered. 

## 3. Results

The group of participants in this study comprised 15 children. Most (nine) were female and the majority of ZikV infections occurred in the second trimester of pregnancy (six cases) (Table 1). Only one participant (participant 14) was born prematurely (33 weeks) and used ototoxic medication (aminoglycoside–vancomycin) in the neonatal ICU. All participants were born with a normal head circumference, but Participant 1 developed late microcephaly and Participant 14 developed late macrocephaly due to hydrocephalus.

As this is a series of cases about medium/long-term outcomes of a poorly investigated health condition, we decided to present the data in detail. Table 2 shows information about neonatal hearing screening, the period of pregnancy when there was infection by ZikV, and the results of the evaluations performed by each participant, except for Participant 15, who had not reached the age of the youngest age group considered for the rest of the sample and will be described in text. Four participants (1, 6, 14, and 15) according to clinical criteria [30] had CZS, which represents 26.7% of the sample; two of whom (13.3%) had extensive CNS malformations. 

Child 15 entered the research at the age of 14 months; in the first collection, they had a delayed language acquisition with a CS of 56 on the Bayley III and auditory alterations: OEAT absent in both ears, impedanciometry was not possible, and the BAEP was morphologically present, but with a hearing threshold of 40 dBNa bilaterally. The second collection of this case was at 18 months. The patient maintained a low performance in the CS language of 47 and similar auditory alterations as found in the previous evaluation, except for the BAEP, for which there was an improvement of 5 dBNa in the threshold, keeping it 35 dBNa below the optimal value. This child was referred to the speech therapy and otorhinolaryngology services since the first exam, and the use of bilateral hearing aids was recommended. However, the parents did not accept the performance of this procedure until the last contact the research team had with them; at that time, the infant was 18 months old. 

Considering all ages evaluated, eight (53.33%) of the fifteen study participants showed altered results in at least one of the hearing tests and seven participants (46.67%) showed a lower-than-expected performance in language in at least one of the assessments in the study period. Four (26.67%) of them simultaneously presented these negative outcomes, with hearing and language changes at the same evaluated age. The age group of 30 to 31 months (nine children) had the largest number of participants with changes (six or 66.67%) in the tests and examinations performed, four (44.44%) had lower than expected language performance, four (44.44%) had alterations in at least one hearing test, and two (22.22%) presented both outcomes simultaneously. Attention is drawn to the cases of Participants 5 and 9. All their hearing tests were altered, thus suggesting a conductive hearing loss and a low performance on the language scale.

The age group of 36 to 38 months had the highest number of participants evaluated (11 children), but proportionally, it had the lowest number of participants with altered results in tests and exams (four or 36.40%): three (27.27%) with alteration in some hearing test; however, there was no language impairment. The age group of 40 to 42 months counted eight children with some altered results in half of them (four); two (25%) showed changes in language acquisition, three (37.50%) showed changes in at least one of the hearing tests, and one (12.50%) showed both outcomes. 

Participants 5 and 9, who had changes in audiological exams at the first assessment, showed improvement in language development after clinical treatment for serous otitis media, performing within normal limits on the Bayley III in the next assessment (36 to 38 months). Participant 8 performed the audiological exams for the first time at 42 months and showed a similar outcome to that of Participants 5 and 9. There were changes in all tests, suggesting a conductive hearing loss accompanied by language impairment. This participant returned at 47 months, after clinical treatment for serous otitis media with an otorhinolaryngologist. They presented normal results in all audiological exams, but it was not possible to apply the Bayley III due to the age limit allowed for comparison with normative data (42 months). However, the parents reported an improvement in their verbal communication and the researchers noticed a greater participation during data collection. 

The association between the BAEP classification, considering it altered when affecting both sides, and the classification in the Bayley III did not show a significant *p*-value in the age groups of which it was possible to perform the analysis (Table 3). However, in all cases, where there was a bilateral alteration in the exam, there was an alteration in language development. The classification of the results of this same exam and the Bayley III were also analyzed using the Mann–Whitney test. There were no significant associations in the age groups to which it was possible to apply the test. However, the median values show a difference of more than 20 points in the composite score between the groups, and in both age groups, the median of the group with altered BAEP was below the cutoff point recommended as a normality criterion (Table 4). 

## 4. Discussion

This study described early and late hearing disorders in 15 children with GZVI, as well as language development and its possible correlation with hearing disorders. Despite the low frequency of early hearing disorders, there was a high prevalence of late changes in audiologic tests. More than half of the participants showed this condition in some of the age groups evaluated, and almost half (46.67%) performed below the expected for the age on the Bayley III in some of the assessments. Considering the studies already published to date, this is the first to report on specific hearing and language outcomes in children older than two years of age in cases where there was ZikV.

In the present sample, four (26.7%) of the fifteen participants fell within CZS. participants 1 and 14 had a similar condition, with central changes in the acquisition of language due to an extensive CNS malformation. In the other two cases, CNS changes were minor, but Participant 15 had peripheral sensorineural hearing impairment and a delayed language development; the other participant (#6) did not show changes in audiological or language tests in studied ages. It is not possible to compare the incidence of CZS in confirmed cases of infection during the gestational period due to the lack of studies that used the same inclusion and exclusion criteria as those of the present study (only cases confirmed by laboratory tests), but it is considered high because it affected more than a quarter of the cases.

As for the presence of auditory alterations in cases of CZS, the present results are in agreement with the cases reported by Abramov et al. [31], since the two children with a greater brain involvement presented all audiologic exams within the normal range and a BAEP with appropriate morphology and thresholds; Participant 15 presented a BAEP with normal morphology and a lowered hearing threshold, suggesting sensorineural hearing loss of peripheral origin.

Considering the sample as a whole, the incidence of conductive hearing disorders was the most prevalent. It occurred in almost half of the participants (46.7%). It is indicative of secretory otitis media (SOM), with the presence of secretion in the middle ear with no signs of acute infection. Considering the age of evaluation of these cases (between 30 to 42 months), the prevalence can be considered high [32]. This outcome was also that reported in the study by Leite et al. [33] and may be indicative of altered immune responses and/or the presence of structural changes in the middle ear, raising the possibility that CZVI is a predisposing factor for this occurrence.

Participants 5, 8, and 9 presented similar results, with changes in all audiological exams of a conductive characteristic and impaired language acquisition. These findings reinforce the need to perform tests that allow the diagnosis of alterations of this type at least until the age of three in populations similar as that of the present study, ensuring the possibility of referral to treatments that enable an appropriate language development, which meets the recommendations of the Joint Committee On Infant Hearing (JCIH) [34]. Children in the first three years of life have a great language development, and secretory otitis media (SOM) is an important factor in altering language acquisition [35,36].

Participants who showed changes in OEAT and immittance testing, but without BAEP involvement, had no language acquisition problems, suggesting that language acquisition is more affected when there is a greater involvement of the middle ear and/or neural pathways. It is worth mentioning that there were language alterations in participants who had all audiological exams altered or extensive CNS malformations, except for Participant 12, in whom these conditions were not present, but who had a delay in language acquisition in the first assessment performed at 30/31 months. It is believed that the case of this participant is related to a family component of extreme care or overprotection [37], since after guidance on this aspect, in the second assessment, language development became appropriate for its age.

To date, no studies have been published on language development and hearing disorders in infants or preschoolers who were exposed to ZikV during pregnancy [15], making it difficult to perceive the real contribution of this health condition to outcomes found in the present sample (high prevalence of SOM and language disorders).

Although there was no significant association between the BAEP results and the Bayley III, it is important to note that in all cases where there was a bilateral alteration in this exam, there was an alteration in language development. This seems to be a good indicator of the risk of delay in language acquisition. In this way, the results of the present study reinforce the idea that that it must be the exam of choice for monitoring cases of GZVI. Its periodic performance is recommended, even with normal BAEP results in the first year of life. The BAEP is the best choice for assessing hearing acuity in children [34] and is able to identify both conductive and sensorineural hearing changes, in addition to making the top diagnosis and indicating the auditory threshold.

As limitations, the fact that not all participants were evaluated in the three age groups stands out, but this is expected due to the longitudinal character of the study. This situation was aggravated by the need to interrupt collections as of March 2020 due to the COVID-19 pandemic. This study design did not enable statistical inferences between hearing disorders and CZVI, but as a strength, it reveals the relevant occurrence of these disorders in the region studied and allows for future studies in older ages of this population, mainly to investigate the first school years.

## 5. Conclusions

The case series of the present study reveals that infants and preschoolers with CZVI may present early neurosensory loss and late hearing loss, with fluctuating character. Most hearing losses were conductive (46.67%); only one case (6%) showed a sensorineural hearing loss. Even if there were no significant association between the audiological exams results and the Bayley III performance, in the present sample, language development was below expectations for the age in the participants who had alterations in the three audiological exams, when there is an early hearing loss or extensive lesions to the CNS.

The results suggest that it is important to perform audiological exams, especially the morphological and auditory threshold BAEP, to monitor cases of CZVI up to the age of three. Prompt referrals for treatments and assessment of language development are recommended in cases when there is a change in the results of these exams.

## Figures and Tables

**Table 1:** Characteristics of the study participants (*n* = 15).

	Number	%
Sex		
Female	9	60.0
Male	6	40.0
Gestational Period of ZikV Infection		
1st Gestation Period	4	26.7
2nd Gestation Period	6	40.0
3rd Gestation Period	4	26.7
Did not know how to report	1	6.7
Marital status		
Single	3	20.0
Married	7	46.7
Stable union	5	33.3
Maternal education level		
Incomplete elementary school	1	6.7
Complete elementary school	1	6.7
Complete High School	6	40.0
Incomplete High School	3	20.0
Complete higher education	3	20.0
Postgraduated	1	6.7
Family socioeconomic class		
A	1	6.7
B2	5	33.3
C1	4	26.7
C2	3	20.0
D–E	2	13.3
	Mean	SD
Gestational Age at Birth (in weeks)	38.80	±2.01
Birth weight (in grams)	2959.07	±629.61
Head circumference at birth (in centimeters)	34.13	±1.64

**Table 2:** Results by age group.

			Screening		Age Range 30–31 Months							Age Range 36–38 Months							Age Range 40–42 Months						
			AOE		BAY	AOE		IMPE		BAEP		BAY	AOE		IMPE		BAEP		BAY	AOE		IMPE		BAEP	
PI.	GA.	HC.	RE	LE	CS	RE	LE	RE	LE	RE	LE	CS	RE	LE	RE	LE	RE	LE	CS	RE	LE	RE	LE	RE	LE
1	1	32			47	nl	nl	A	A	nl	nl	47	nl	nl	A	A	nl	nl							
2	2	33	nl	nl	112	nl	nl	A	A	nl	nl	106	nl	nl	A	A	nl	nl							
3	1	33	nl	nl								115	nl	nl	A	C	nl	nl	115	nl	nl	A	A	nl	nl
4	3	35	nl	nl	100	nl	nl					100	nl	nl	A	A	nl	nl	103	nl	nl	A	A	nl	nl
5	1	37	nl	nl	71	alt	alt	B	C	cd	cd	89	nl	nl	A	A	nl	nl							
6	1	35	nl	nl	103	nl	nl	A	A	nl	nl	106							100	nl	nl	A	A	nl	nl
7	2	34	nl	nl															109	nl	alt	A	C	nl	nl
8	3	33	nl	nl															83	alt	alt	C	C	cd	cd
9	1	37			83	alt	alt	C	C	cd	cd	91	alt	nl	A	C	nl	nl							
10	2	36	nl	nl	124	nl	nl	C	C	nl	nl	118	nl	nl	C	C	nl	nl	91	nl	nl	C	C	nl	nl
11	2	34	nl	nl									nl	nl	A	A	nl	nl							
12	2	35	nl	nl	65	nl	nl	A	A	nl	nl	97	nl	nl	A	A	nl	nl							
13	3	33	nl	nl	121	alt	alt	B	A	nl	nl	118	nl	nl	A	A	nl	nl	103	nl	nl	A	A	nl	nl
14	2	32																	47	nl	nl	A	A	nl	nl

Caption: PI = participant identification; GA = gestational age, HC = head circumference, nl = normal, alt = altered, cd = conductive, RE = right ear, LE = left ear, CS = composite score, BAY = Bayley III, AOE = acoustic otoemissions, IMPE = impedanciometry, BAEP = brainstem auditory evoked potential, A = curve A—normal, B = curve B—serous otitis media, C = curve C—tubal dysfunction. 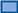 normal exams; 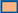 altered exams.

**Table 3:** Association between the BAEP variable (considering two sides) and Bayley III (composite score—CS).

		Bayley III (CS)				Total (%)	*p*-Value
		Normal		Altered			
			f	(%)	f		
BAEP (30–31 m)	Normal	4	(66.7)	2	(33.3)	100	0.214 *
	Altered	0	(0)	2	(100)	100	
	Total	4	(50.0)	4	(50.0)	100	
BAEP (36–38 m)	Normal	8	(88.9)	1	(11.1)	100	a
	Altered	0	(0)	0	(0)	0	
	Total	8	(88.9)	1	(11.1)	100	
BAEP (40–42 m)	Normal	6	(85.7)	1	(14.3)	100	0.250 *
	Altered	0	(0)	1	(100)	100	
	Total	6	(75.0)	2	(25.0)	100	

* Fisher’s exact test; *f* = frequency, a—no statistical analysis was performed because BAEP 36 m–38 m was considered a constant.

**Table 4:** Comparison * between the BAEP groups (considering two sides) and Bayley III (composite score—CS).

Variable	Min	Mean ± SD	Med	Max	*p*-Value
BAEP (30–31 m)					0.64
Normal group	47	95.33 ± 31.85	107.50	124	
Altered group	71	77 ± 8.42	77	83	
BAEP (36–38 m)					a
Normal group	47	97.89 ± 22.05	100	118	
Altered group					
BAEP (40–42 m)					0.50
Normal group	47	95.43 ± 22.61	103	115	
Altered group	83	83	83	83	

Caption: min = minimum; SD = standard deviation; med = median; max = maximum. * Mann–Whitney test. a—no statistical analysis was performed because BAEP 36 m–38 m was considered a constant.

## Data Availability

The data presented in this study are available on request from the corresponding author. The data are not publicly available due to patient confidentiality.
